# Supplementary material for: Strawberry FaWRKY25 Transcription Factor Negatively Regulated the Resistance of Strawberry Fruits to Botrytis cinerea
Source: Genes (Basel). 2020 Dec 31;12(1):56. doi: 10.3390/genes12010056 (PMC7824073; doi:10.3390/genes12010056)

Supplemental Material

Strawberry *FaWRKY25* transcription factor negatively regulated the resistance of strawberry fruits to *Botrytis cinerea* by suppressing the jasmonic acid pathway

Sizhen Jia, Yuanhua Wang , Geng Zhang, Zhiming Yan, and Qinsheng Cai[*]

[*]Correspondence:

Qingsheng Cai:

qscai@njau.edu.cn

1 SUPPLEMENTARY DATA

Supplementary Tables S1,S2&S3

Supplementary Figure S1, S2&S3

Supplementary Table S1. Primers used for vector construction. The restriction enzyme sites in the primer sequences are underlined.

Gene	Forward primer (5'→3')	Reverse primer (5'→3')
FaWRKY25 Over-expression	ATGGCTTCCTCTTCCGGGAGTCTAGA	TCAACATAGCAACGACTCGAAGAA
FaWRKY25-P	<u>GGCGCGCCA</u>GCCAGAGTGTTAAAAAGGAGCACA AscI restriction enzyme sites	<u>ATTTAAAT</u>CGATTCCCTTCACTTGTTTCTGGC SwaI restriction enzyme sites
FaWRKY25-N	<u>TTAATTAA</u>GCCAGAGTGTTAAAAAGGAGCACA PacI restriction enzyme sites	<u>CCTAGGG</u> CGATTCCCTTCACTTGTTTCTGGC AvrII restriction enzyme sites
FaWRKY25 RNAi	TTACCATGGGGCGCGCCCAATCGATGATTT	GCAGGACTCTAGGGACTAGTCCCGGGTCTT

Supplementary Table S2. Quantitative PCR primers used to analyze the expression of resistance-related gene in strawberry fruits.

Gene	Forward (5'→3')	Reverse (5'→3')	Accession No.
FaLOX	ATCTGAAGGTGAGTGAGAGC	GTGCCCAAGAACATCATCTA	AJ578035.1
FaAOS	CAGAACCCTCTCCTATCTCG	GATTGTGACACGCTTCGTC	XM004291875
FaOPR2	GGAGCCGTTATTTTCTGTCAGC	TCAAAACCTGCTCGAATGGC	XM004294463.2
FaOPR3	GAAGCCACTGGAGTTTCTG	AGAATACACCACCTTTAGCAT	XM004287577.2
FaJAR1	GGTGTGCCATTGGTTAGTGC	CACCCCAATCCAGCTCTCAG	XM004306646
FaJAR2	ATGGAGCCGGTGTGCAATAA	GTTCCAGCGGTTAGTCCTCC	XM011470645
FaCOI1	GAGGAGATTGCGGAGTCGTT	CGGAGCACTTGTCCAGCTTA	XM004307565
FaMYC2	CTCTCCGAGCTGTTGTTCCA	TCTGACTCCGTCGTTTGCAG	XM004300191
FaJAZ1	AACCTCGTCGACATGCAGTT	ATTTGAAGCCTCTGGGAGCC	XM004287607
FaJAZ4	AGAAGTGCTGGTGCACATTG	TGGGCATAAATCTGGAGGAC	XM004303663
FaJAZ5	CACCATGAACTTGCTCAACG	GAAAGGTCGCTGAAGACGAG	XM011464878
FaJAZ8	ATTTGGAGCTCCGCCTTAAT	GAAGATCGTCAATGGCTGCT	XM004293578
FaJAZ10	TTCCAGAAGTTCCTCGAACG	GATTTCCTGGCTGCAATCAC	XM004310081
FaJAZ12	GAAGCGTAGGGACAGATTGG	AACCGGAAGAAGCATCATTG	XM011462941
FaWRKY1	TGTGCAGGGTGTGCTCTCTT	TGGGATTTGGATTGCCTTTTAC	XM004293406
FaWRKY2	TGCTGGACTCTCCTGTCTTCC	CTGGGAAAAGTTCGCTTGCT	XM004294821
FaWRKY11	ATCCAGAAATCCCAAAGCAAAC	GGTGGTGACGGAAGAAAGAAAC	XM004299750
FaWRKY33	ACCGTTCAACTGAGCAACAAGA	AAGAAGGGGAAGGAGGAGGAG	XM004302509
FaWRKY25	CGGATCATTCTTTCGGCTCT	CCCTCCTCCTGACTTGCTTCT	XM004294710
FaWRKY40	TGCTGATAAGACCTGTGAAAATGAG	GTTCTAATGGAGCGGGCAAC	XM004291907
FaWRKY57	CGTGGTCGGATCTGTCTCCT	ATTCGCTTCTGCCCCTTCTT	XM011464727
FaWRKY70	GGGCGTCAAGGAAGAAGAGA	CGGACGACTCAAGCACACA	XM004305031
FaWRKY75	ACGACGACCATTATTCCGATG	CATACTTGGGCTTTCTCGTTTTCT	XM004304482
FaBG2-1	CTAAATATCTTCTTCCTGCCATA	AATGTTGTATCTATTGCTGTTG	AY170375
FaBG2-2	ACCGGGACTCCCAAGAGACCAAATG	TGTGAGCCTGCACTAGCCAAAGGTG	AY989818
FaBG2-3	TCCGAGAGTGGTTGGCCATCTGAAG	TCCATTTGGTCTCTTCGGAGTCCCG	AY989819
FaPGIP1	TGCTAGAATTCGATCTGTCCAAGG	ATTATCCAATTGGGTCAACTGCTC	EU117215
FaPGIP2	TCCTCATGGAAATCCGACGCCGAC	TCCTCATGGAAATCCGACGCCGAC	EU117213
FaCHI2-2	GCACAACAGGTGATGTTGC	GTAATGACGTCGTGGCTTGA	AF320111
FaCHI3-1	AGGTCTTCTTAGGACTCCCTG	CTTGGACCAAAGCATGACACCGCC	AF134347
FaActin	GCCAACCGTGAGAAGATG	TCCAGAGTCAAGAACAATACCAG	XM011469959

Supplementary Table S3. Comparison of the fruit tissue damage levels in *FaMAPK25*-OE and *FaMAPK25*-RNAi strawberry fruits after *Botrytis cinerea* inoculation.

The severity of tissue damage was evaluated at four levels according to the system described by Zhang et al. (2020): I, weakly visible lesion (up to 10 % fruit damage); II, moderate lesion (10 %–25 % fruit damage); III, enlarged lesion (25 %–50 % fruit damage); IV, very affected fruit (>50 % fruit damage). The experiment was repeated three times, and 60 fruits were injected in each batch for each vector.

Treatment	DABI-1					DABI -2			
	I	II	III	IV		I	II	III	IV
WT	**0**	**0**	**0**	**0**		**0**	**0**	**0**	**0**
EV-OE	**0**	**0**	**0**	**0**		**0**	**0**	**0**	**0**
FaWRKY25-OE	**0**	**0**	**0**	**0**		**0**	**0**	**0**	**0**
EV-RNAi	**0**	**0**	**0**	**0**		**0**	**0**	**0**	**0**
FaWRKY25- RNAi	**0**	**0**	**0**	**0**		**0**	**0**	**0**	**0**
	DABI -3					DABI -4			
	I	II	III	IV		I	II	III	IV
WT	**0**	**0**	**0**	**0**		**7.1d**	**0**	**0**	**0**
EV-OE	**0**	**0**	**0**	**0**		**75.2a**	**6.5b**	**0**	**0**
FaWRKY25-OE	**36.5**	**4.3**	**0**	**0**		**58.5c**	**34.6a**	**6.9**	**0**
EV-RNAi	**0**	**0**	**0**	**0**		**62.6b**	**4.1b**	**0**	**0**
FaWRKY25- RNAi	**0**	**0**	**0**	**0**		**3.1d**	**0**	**0**	**0**
	DABI -5					DABI -6			
	I	II	III	IV		I	II	III	IV
WT	**46.5b**	**41.8b**	**11.7c**	**0**		**8.2c**	**23.3b**	**68.5b**	
EV-OE	**25.9d**	**54.3a**	**19.8b**	**0**		**8.9c**	**14.1c**	**77.0a**	
FaWRKY25-OE	**0**	**7.6c**	**76.3a**	**16.1**		**0**	**0**	**10.4c**	**89.6**
EV-RNAi	**33.2c**	**58.7a**	**8.1c**	**0**		**10.9b**	**18.5c**	**70.6b**	**0**
FaWRKY25- RNAi	**74.1a**	**35.9b**	**0**	**0**		**37.9a**	**62.1a**	**0**	**0**

Supplementary Figure S1. Nucleotide sequence and amino acid sequence of *FaWRKY25* gene

```
              10        20        30        40        50        60
1     ATGGCTTCCTCTTCCGGGAGTCTAGACACCTCAGCGAATTCCCACCCCAGCAACTTCACC
1      M  A  S  S  S  G  S  L  D  T  S  A  N  S  H  P  S  N  F  T

              70        80        90       100       110       120
61    TTCTCCACTCACCCCTTCATGAACACCTCCTTCTCCGACCTCCTATCCTCCGGCGCCGAA
21     F  S  T  H  P  F  M  N  T  S  F  S  D  L  L  S  S  G  A  E

             130       140       150       160       170       180
121   CCCACAAACCCAATAACCCGATACTCCGGGCACGGGCTATCCGACCGTATTGCGGAGCGG
41     P  T  N  P  I  T  R  Y  S  G  H  G  L  S  D  R  I  A  E  R

             190       200       210       220       230       240
181   ACCGGGTCAGGTGTCCCCAAGTTCAAGTCACTCCCGCCTCCCCAGCTCCCCATTTCGCCT
61     T  G  S  G  V  P  K  F  K  S  L  P  P  P  Q  L  P  I  S  P

             250       260       270       280       290       300
241   CCCTCGGTCTCTCCCTCTTCCTACTTCGCAATCCCGCCTGGGATGAGCCCTGCCGAGCTT
81     P  S  V  S  P  S  S  Y  F  A  I  P  P  G  M  S  P  A  E  L

             310       320       330       340       350       360
301   CTCGACTCTCCTGTACTTCTCAGCTCCTCCAACATTCTTCCATCTCCAACTACTGGAGT
101    L  D  S  P  V  L  L  S  S  S  N  I  L  P  S  P  T  T  G  S

             370       380       390       400       410       420
361   TTTGCAGCTCAAGCCTTTAATTGGAGAACCAATTCTGGTTCTGGCACTAACAACAACAAT
121    F  A  A  Q  A  F  N  W  R  T  N  S  G  S  G  T  N  N  N  N

             430       440       450       460       470       480
421   AACAACAACAGCCAGAGTGTTAAAAAGGAGCACAAGAACTACTCTGATTTCTCTTTCCAA
141    N  N  N  S  Q  S  V  K  K  E  H  K  N  Y  S  D  F  S  F  Q

             490       500       510       520       530       540
481   ACCCAAACCAGGCCCCCTCCAACTTCATCATCAGCAATTTTCCAATCCTCAAACAGCTCA
161    T  Q  T  R  P  P  P  T  S  S  S  A  I  F  Q  S  S  N  S  S

             550       560       570       580       590       600
541   ATTCAGACTGCTCAAGAACAGGCATGGAATAACTATCTAGGTCCTTCCAAACAAGATGAG
181    I  Q  T  A  Q  E  Q  A  W  N  N  Y  L  G  P  S  K  Q  D  E

             610       620       630       640       650       660
601   TTTTCTTCAGGGAAGAGTATGGTGAAACCTGAGTATGGTTCTTCAGTGCAGAGCTTCTCT
201    F  S  S  G  K  S  M  V  K  P  E  Y  G  S  S  V  Q  S  F  S

             670       680       690       700       710       720
661   TCTGAAATGGCCACCAATATGCAAGGCAATAATGTTCAGGCCAACAGCAATGGTGGTTTC
221    S  E  M  A  T  N  M  Q  G  N  N  V  Q  A  N  S  N  G  G  F

             730       740       750       760       770       780
721   CAATCAGATCAGTACAGCAACTACAATAACACCAACACCACCGGCAACAACAACAACAAC
241    Q  S  D  Q  Y  S  N  Y  N  N  T  N  T  T  G  N  N  N  N  N

             790       800       810       820       830       840
781   AACAACACCCAGCTCCAGTCTCATCAGTGCTTGAACAAGAGATCAGATGATGGGTTCAAT
261    N  N  T  Q  L  Q  S  H  Q  C  L  N  K  R  S  D  D  G  F  N

             850       860       870       880       890       900
841   TGGAGGAAATATGGCCAGAAACAAGTGAAGGGAAGCGAAAATCCGCGAAGCTATTACAAG
281    W  R  K  Y  G  Q  K  Q  V  K  G  S  E  N  P  R  S  Y  Y  K

             910       920       930       940       950       960
901   TGTACTTATCCGAATTGCCCAACCAAGAAAAAGGTTGAGAGGTCTTTGGATGGACAAATC
301    C  T  Y  P  N  C  P  T  K  K  K  V  E  R  S  L  D  G  Q  I

             970       980       990      1000      1010      1020
961   ACCGAGATAGTTTACAAGGGTAATCATAACCATCCCAAGCCACAGAATACTAGAAGGTCA
321    T  E  I  V  Y  K  G  N  H  N  H  P  K  P  Q  N  T  R  R  S

            1030      1040      1050      1060      1070      1080
1021  GGTTCATCAGCGGCTTCTACCCATGCCGTTCAGGTTTCAAGTCAAACCACCAATGACATC
341    G  S  S  A  A  S  T  H  A  V  Q  V  S  S  Q  T  T  N  D  I

            1090      1100      1110      1120      1130      1140
1081  CCGGATCATTCTTTCGGCTCTCATGGGAATTCACAAATGGATAACTCCATTGGTACACCA
361    P  D  H  S  F  G  S  H  G  N  S  Q  M  D  N  S  I  G  T  P

            1150      1160      1170      1180      1190      1200
1141  GAGAATTCATCCCTTTCACTGGGAGATGATGAGTTTGATCAGAGCTCTCAGAGAAGCAAG
381    E  N  S  S  L  S  L  G  D  D  E  F  D  Q  S  S  Q  R  S  K

            1210      1220      1230      1240      1250      1260
1201  TCAGGAGGAGGGGATGAGTATGATGAAGATGAGCCTAATGCCAAAAGATGGAAAAAAGAA
401    S  G  G  G  D  E  Y  D  E  D  E  P  N  A  K  R  W  K  K  E

            1270      1280      1290      1300      1310      1320
1261  GGTGACAATGAATGTATATCAGCCCCTGGAAGTAGAACAGTAAGAGAACCAAGAGTTGTA
421    G  D  N  E  C  I  S  A  P  G  S  R  T  V  R  E  P  R  V  V

            1330      1340      1350      1360      1370      1380
1321  GTTCAAACAACTAGTGACATTGATATTCTTGATGATGGGTATAGGTGGAGGAAGTATGGT
441    V  Q  T  T  S  D  I  D  I  L  D  D  G  Y  R  W  R  K  Y  G

            1390      1400      1410      1420      1430      1440
1381  CAGAAAGTAGTAAAGGGCAACCCAAATCCAAGGAGCTACTACAAGTGCACAAATCCAGGC
461    Q  K  V  V  K  G  N  P  N  P  R  S  Y  Y  K  C  T  N  P  G

            1450      1460      1470      1480      1490      1500
1441  TGTCCAGTGAGGAAGCACGTAGAGCGAGCTTCACATGATCTTAGAGCAGTGATCACAACC
481    C  P  V  R  K  H  V  E  R  A  S  H  D  L  R  A  V  I  T  T

            1510      1520      1530      1540      1550      1560
1501  TACGAAGGGAAGCACAACCACGATGTTCCTGCAGCTCGTGGCAGTGGCTCTGCCAACAGG
501    Y  E  G  K  H  N  H  D  V  P  A  A  R  G  S  G  S  A  N  R

            1570      1580      1590      1600      1610      1620
1561  CCTTTACCAAGCAACAATACCAACAACAACAACAACATGAACAACCACCACAATGTAGCC
521    P  L  P  S  N  N  T  N  N  N  N  N  M  N  N  H  H  N  V  A

            1630      1640      1650      1660      1670      1680
1621  CCAGCAATGAGGCCTATGACCCATCACAGTACTAACAATGCTGCTAACAACAACCCTATT
541    P  A  M  R  P  M  T  H  H  S  T  N  N  A  A  N  N  N  P  I

            1690      1700      1710      1720      1730      1740
1681  CAAAATCTAAGGCTACCGACATCAGAAGGGCAAGCACCCTTTACTCTCGAGATGTTGCAG
561    Q  N  L  R  L  P  T  S  E  G  Q  A  P  F  T  L  E  M  L  Q

            1750      1760      1770      1780      1790      1800
1741  AGTCCAGGAAGTTACGGATTTGCAGGGTTTGAGAACTCCATGGGGTCATACATGAACCAA
581    S  P  G  S  Y  G  F  A  G  F  E  N  S  M  G  S  Y  M  N  Q

            1810      1820      1830      1840      1850      1860
1801  ACACACCTCAATCACAACAACATGTTTTCTAAAACCAAGGATGAGCCCAGAGATGATGCA
601    T  Q  L  N  D  N  N  M  F  S  K  T  K  D  E  P  R  D  D  A

            1870      1880
1861  TTCTTCGAGTCGTTGCTATGTTGA
621    F  F  E  S  L  L  C  *
```

Supplementary Figure S2. Sequence alignment of the deduced amino acid sequences of WRKY25.

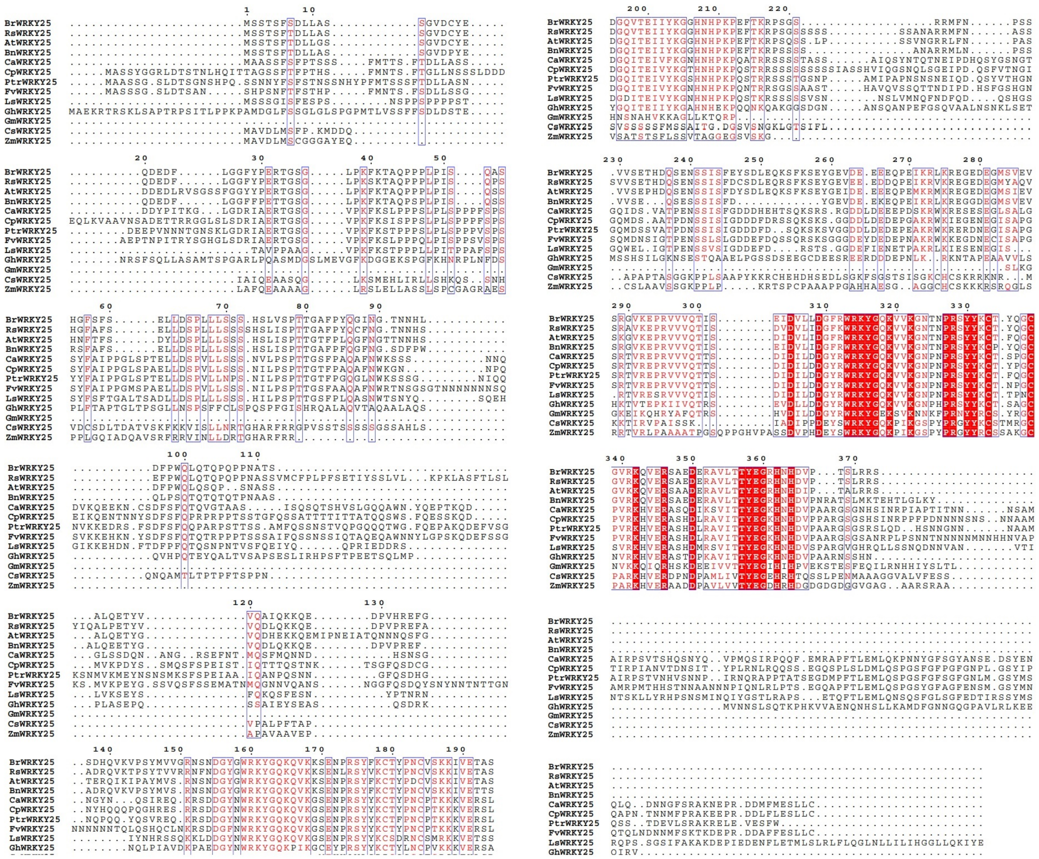

Suppl

cinere

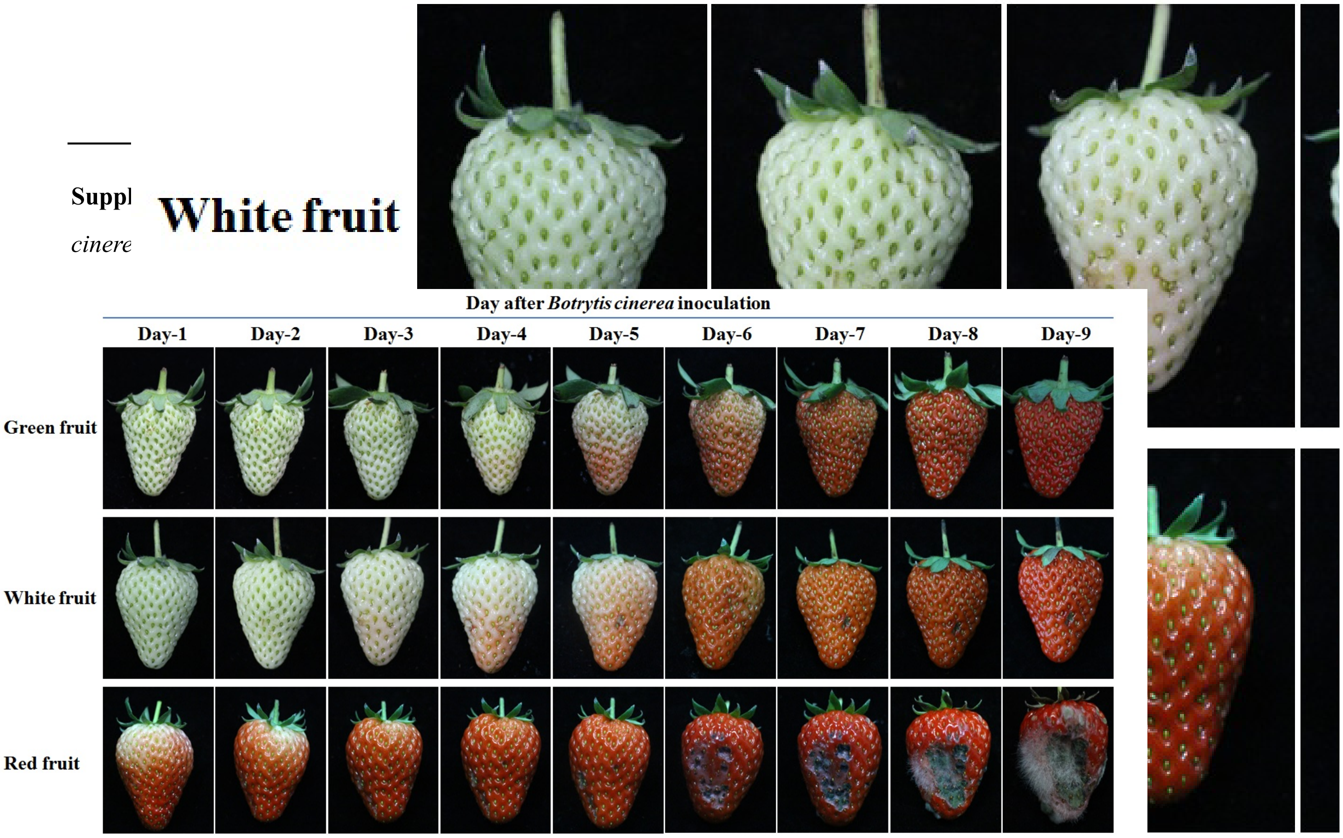

Supplement: Supplementary file 1 [file genes-12-00056-s001.pdf]
